# Cancer Stem Cell Marker *Musashi-1* rs2522137 Genotype Is Associated with an Increased Risk of Lung Cancer

**DOI:** 10.1371/journal.pone.0095915

**Published:** 2014-05-02

**Authors:** Xu Wang, Ji-Fan Hu, Yehui Tan, Jiuwei Cui, Guanjun Wang, Randall J. Mrsny, Wei Li

**Affiliations:** 1 Cancer and Stem Cell Center, First Affiliated Hospital, Jilin University, Changchun, Jilin, P.R. China; 2 Stanford University Medical School, VA Palo Alto Health Care System, Palo Alto, California, United States of America; 3 Department of Pharmacy and Pharmacology, University of Bath, Bath, England; Geisel School of Medicine at Dartmouth College, United States of America

## Abstract

Gene single nucleotide polymorphisms (SNPs) have been extensively studied in association with development and prognosis of various malignancies. However, the potential role of genetic polymorphisms of cancer stem cell (CSC) marker genes with respect to cancer risk has not been examined. We conducted a case-control study involving a total of 1000 subjects (500 lung cancer patients and 500 age-matched cancer-free controls) from northeastern China. Lung cancer risk was analyzed in a logistic regression model in association with genotypes of four lung CSC marker genes (CD133, ALDH1, *Musashi-1*, and EpCAM). Using univariate analysis, the *Musashi-1* rs2522137 GG genotype was found to be associated with a higher incidence of lung cancer compared with the TT genotype. No significant associations were observed for gene variants of CD133, ALDH1, or EpCAM. In multivariate analysis, *Musashi-1* rs2522137 was still significantly associated with lung cancer when environmental and lifestyle factors were incorporated in the model, including lower BMI; family history of cancer; prior diagnosis of chronic obstructive pulmonary disease, pneumonia, or pulmonary tuberculosis; occupational exposure to pesticide; occupational exposure to gasoline or diesel fuel; heavier smoking; and exposure to heavy cooking emissions. The value of the area under the receiver-operating characteristic (ROC) curve (AUC) was 0.7686. To our knowledge, this is the first report to show an association between a *Musashi-1* genotype and lung cancer risk. Further, the prediction model in this study may be useful in determining individuals with high risk of lung cancer.

## Introduction

Lung cancer is one of the most commonly diagnosed malignancies and the leading cause of cancer-related death in the world [1]. Cigarette smoking is considered as an important risk factor for lung cancer. However, only 10–15% of smokers develop lung cancer, suggesting that individual variation in genetic susceptibility to lung cancer in the general population may play a role. Cancer stem cells (CSCs) are a small minority of cells in a heterogeneous tumor population that drives tumor growth and have been associated with resistance to chemo- and radiation-therapies [2]–[4]. It has been demonstrated that lung CSCs play an important role in tumor initiation [5], [6]. CDCs also share some similarities with normal stem cells, including self-renewal and differentiation, in addition to their potent tumor-driving capability [7]–[10]. CSCs are characterized by expression of particular molecular markers that play an important role in promoting stem cell self-renewal and maintenance [11].

Aberrant expression of CSC markers is associated with the initiation and development of lung cancer, including cluster of differentiation 133 (CD133), Musashi RNA-binding protein 1 (*Musashi-1*), aldehyde dehydrogenase 1 (ALDH1), epithelial cell adhesion molecule (EpCAM), B-cell-specific moloney murine leukemia virus integration site 1 (Bmi-1), Octarner binding factor 4 (OCT-4), and Glycine dehydrogenase (GLDC) [12]–[20]. CD133, initially described as a surface antigen specific for human hematopoietic stem cells [21], [22], is now being used as an isolation marker of CSCs from lung cancer [23]. *Musashi-1*, an RNA-binding protein, is expressed in various epithelial stem cells and plays an important role in regulating the maintenance and differentiation of stem/precursor cells [24]–[26]. *Musashi-1* is over-expressed in several tumor tissues, including lung cancer [17], gliomas [27], intestinal adenomas [28], [29] and hepatomas [30], suggesting a correlation with oncogenic development. ALDH1 is widely regarded as a surface marker of CSCs in lung cancer [31]–[33]. ALDH positive lung cancer stem-like cells have longer telomeres than the non-CSC cells [34]. EpCAM, a type I transmembrane glycoprotein of ∼40 kDa, is overexpressed in a variety of epithelial tumors, including lung cancer [18]–[20]. EpCAM is involved in intercellular adhesion and interacts with E-cadherin to induce cell adhesion [35]. Overexpression of EpCAM is linked directly to stimulation of the cell cycle and proliferation by upregulating c-myc and cyclin A/E [36]. Inhibition of EpCAM by small inhibitory RNA diminishes cell proliferation, migration and invasiveness [37].

Together, these observations have correlated aberrant function of CSC marker molecules with cellular hallmarks of cancer: hyperproliferation and metastatic behaviors. While SNPs have been extensively studied for their association with the risk and prognosis of cancers, little is known about the potential role of SNPs in CSC marker genes with relation to cancer. In this study, we examined the association of lung cancer risk in a Chinese population with polymorphisms of the well-established CSC marker genes CD133, ALDH1, *Musashi-1* and EpCAM. A forecasting model was constructed using CSC marker SNPs and epidemiologic factors; the results provide a novel method to predict individuals at increased risk of developing lung cancer.

## Methods

### Study population

We conducted a hospital-based, case-control study involving a total of 1000 subjects from northeastern China (Changchun City, Jilin province). All subjects were local residents of Han descent, consisting of 500 patients clinically diagnosed with lung cancer and 500 cancer-free controls. Patients had histologically-confirmed primary lung cancer without previous cancer history, did not receive radiotherapy, chemotherapy or other anti-cancer therapy. Controls were randomly selected normal individuals receiving routine physical examinations in the same hospital. Case matching was performed based on age, gender and place of residence. The study was approved by the Ethics Committee of the First Hospital of Jilin Medical University, and conducted according to the Declaration of Helsinki Principles. All subjects were provided written informed consent.

### Diagnostic criteria and Data Collection

A standardized interview was conducted by trained interviewers in the hospital or at the homes of participating individuals. Information regarding socio-demographic details, medical history, family history, lifestyle history, and cancer diagnosis was recorded. Risk factor information and peripheral blood lymphocytes were collected at the time of diagnosis for cancer patients or on the day of interview for controls.

### CSC marker gene polymorphism selection

We used a candidate gene approach [38]–[40] to select SNPs for this study. Four well-established CSC marker genes (CD133, ALDH1, *Musashi-1* and EpCAM) were selected in the study design. Expression of these four proteins had been reported as a marker to identify lung CSCs [41], [42].

Three predefined criteria were used for CSC SNP selection: (a) minor allele frequency (MAF) ≥5% in the HapMap CHB population; (b) SNPinfo website (http://snpinfo.niehs.nih.gov) for candidate CSC gene SNP selection, and (c) publications showing clinical correlations with cancer risk/outcome or recurrence. Using these criteria, five CSC candidate SNPs were chosen in our model analysis: Rs2286455 in the CD133 gene, rs1342024 and rs13959 in the ALDH1 gene, rs2522137 in the *Musashi-1* gene, and rs17036526 in the EpCAM gene (**Table 1**). Based upon literature information, we excluded polymorphisms previously implicated in COPD or lung cancer. Additionally, we did not select SNPs in genes encoding proteins involved in pathways of cell-cycle control, oxidant response, apoptosis and airways inflammation. Finally, we avoided SNPs known to have either functional effects on in vitro assays, or were non-synonymous or in regulatory regions.

**Table:** **Table 1.** Single nucleotide polymorphisms in cancer stem cell marker genes.

Gene	SNP	Base exchange	Gene location	Reference
CD133	rs2286455	G>A	Splice Site	nd
ALDH1A1	rs1342024	G>C	Upstream	[54]
	rs13959	C>T	Synonymous coding	nd
MSI-1	rs2522137	T>G	3′ UTR	nd
EpCAM	rs17036526	G>C	Splice site	nd

UTR: untranslated region; nd: no data.

### Genotyping and quality control

Genomic DNA was isolated from peripheral blood lymphocytes. MassArray (Sequenom, San Diego, CA) was used to genotype CSC markers using allele specific MALDI–TOF mass spectrometry. Primers and multiplex reactions were designed using the RealSNP.com Website. Concordance among the 3 genomic control DNA samples present in duplicate was 100%. Of the SNPs with genotyping data, the sample call rates were more than 95%.

### Statistical analysis

The Hardy-Weinberg equilibrium (HWE) was tested by a best fit chi-square (χ^2^) test that compared expected genotype frequencies with observed genotype frequencies in cancer-free controls. The model was also used to determine the presence of significant differences in genotype and allele distribution as well as SNP frequency between clinically diagnosed lung cancer and controls. A logistic regression model was used to identify independent risk factors for lung cancer. The forward stepwise likelihood ratio method was employed to screen variables in model selection, where the cut-off for variables in the model was 0.05 and the cut-off for variables outside of model was 0.10; an optimal model with minimum akaike information criterion was selected. All categorical variables were set as dummy variables, and the first category of each variable was selected as baseline. The classification ability of the model was evaluated using the area under the receiver operating characteristic (ROC) curve (AUC), and the optimal operating point (OPP) was given afterwards. All analyses were conducted using SPSS v19.0 software (SPSS, Inc., Chicago, IL, USA). All P-values were two-sided, and P-values <0.05 were considered statistically significant.

## Results

### Distribution of genotype and its characteristics in cancer and control populations

We recruited 500 cases of lung cancer and 500 cancer free controls between 2010 and 2012. **Table 2** shows the distribution and frequency of study-specific risk factors between cancer patients and controls.

**Table 2:** Distribution of CSC marker genotypes and characteristics of the case group and the healthy control group.

Characteristic		Case group	Control group
		(n = 500)	(n = 500 )
rs2286455	AA	46 (9.2%)	37 (7.4%)
	GG	266 (53.2%)	262 (52.4%)
	GA	188 (37.6%)	201 (40.2%)
rs1342024	GG	97 (19.4%)	92 (18.4%)
	CC	164 (32.8%)	166 (33.2%)
	GC	239 (47.8%)	242 (48.4%)
rs13959	TT	89 (17.8%)	99 (19.8%)
	CC	176 (35.2%)	159 (31.8%)
	CT	235 (47.0%)	242 (48.4%)
rs2522137	TT	219 (43.8%)	219 (43.8%)
	GG	67 (13.4%)	39 (7.8%)
	GT	214 (42.8%)	242 (48.4%)
rs17036526	GG	224 (44.8%)	233 (46.6%)
	CC	55 (11.0%)	65 (13.0%)
	CG	221 (44.2%)	202 (40.4%)
Gender	male	305 (61%)	302 (60.4%)
	female	195 (39%)	198 (39.6%)
Age	<30	2 (0.4%)	5 (1.0%)
	30–39	14 (2.8%)	16 (3.2%)
	40–49	64 (12.8%)	70 (14.0%)
	50–59	176 (35.2%)	196 (39.2%)
	60–69	174 (34.8%)	148 (19.7%)
	≥70	70 (14.0%)	65 (13.0%)
Education	Junior high school and lower	318 (63.6%)	130 (26.0%)
	High school	97 (19.4%)	144 (28.8%)
	Greater than high school	85 (17.0%)	226 (45.2%)
Smoking	Pack years	14.25 (0–36.0)	0.0 (0.0–6.9)
exposure to	Absent	398 (79.6%)	473 (94.6%)
Pesticide	Present	102 (20.4%)	27 (5.4%)
exposure to	Absent	487 (97.4%)	496 (99.2%)
Gasoline/diesel	Present	13 (2.6%)	4 (0.8%)
exposure to Ink	Absent	493 (98.6%)	497 (99.4%)
	Present	7 (1.4%)	3 (0.6%)
Cooking emissions	Absent	244 (48.8%)	250 (50.0%)
(Total dish-years)	≤50	149 (29.8%))	152 (30.4%)
	51–100	61 (12.2%)	80 (16.0%)
	101–150	46 (9.2%)	18 (3.6%)
Pneumonia	History Absent	477 (95.4%)	490 (98.0%)
	History Present	23 (4.6%)	10 (2.0%)
COPD	History Absent	449 (89.8%)	489 (97.8%)
	History Present	51 (10.2%)	11 (2.2%)
Pulmonary	History Absent	470 (94.0%)	486 (97.2%)
tuberculosis	History Present	30 (6.0%)	14 (2.8%)
Bronchial asthma	History Absent	488 (97.6%)	495 (99.0%)
	History Present	12 (2.4%)	5 (1.0%)
Family history	History Absent	330 (66.0%)	397 (79.4%)
of cancer	History Present	170 (34.0%)	103 (20.6%)
BMI	<18.5	49 (9.8%)	15 (3.0%)
(kg/m^2^)	18.5–24	302 (60.4%)	230 (46.0%)
	≥24	149 (29.8%)	255 (51.0%)

### Association of CSC marker gene SNPs with lung cancer risk in univariate analysis

We first evaluated lung cancer risk using univariate analysis. Among CSC marker gene SNPs selected, the *Musashi-1* rs2522137 GG genotype had a tendency toward a higher incidence of lung cancer than the rs2522137 GG genotype in both recessive model (P = 0.004) and additive model. However, no significant differences were noticed for SNPs in other CSC marker genes in dominant, recessive, additive, or multiplicative models (**Table 3**).

**Table 3:** Association of SNPs with lung cancer risk in univariate analysis.

	Genotype	Univariate OR (95%CI)	P value
rs17036526			
Recessive model	CC + GC	1	0.568
	GG	0.930 (0.725–1.193)	
Dominant model	CC	1	0.331
	GG + GC	1.209 (0.825–1.773)	
Additive model	CC	1	
	GC	1.293 (0.861–1.942)	0.216
	GG	1.136 (0.759–1.700)	0.535
Multiplicative model	G allele	1.004 (0.837–1.205)	0.963
rs2522137			
Dominant model	TT	1	1.000
	GG + TG	1.000 (0.779–1.284)	
Recessive model	TT + TG	1	0.004
	GG	1.829 (1.207–2.733)	
Additive model	TT	1	
	TG	0.884 (0.680–1.150)	0.359
	GG	1.718 (1.110–2.6591)	0.015
Multiplicativemodel	G allele	1.138 (0.942–1.374)	0.179
rs2286455			
Recessive model	GG + GA	1	0.303
	AA	1.268 (0.807–1.992)	
Dominant model	GG	1	0.800
	AA + GA	0.968 (0.755–1.241)	
Additive model	GG	1	
	GA	0.921 (0.709–1.197)	0.540
	AA	1.225 (0.769–1.950)	0.393
Multiplicative model	A allele	0.963 (0.803–1.153)	0.806
rs1342024			
Recessive model	CC + CG	1	0.686
	GG	1.067 (0.778–1.465)	
Dominant model	CC	1	0.893
	GG + CG	1.018 (0.782–1.325)	
Additive model	CC	1	
	CG	1.000 (0.755–1.323)	0.998
	GG	1.067 (0.746–1.526)	0.722
Multiplicative model	G allele	1.028 (0.863–1.226)	0.754
rs13959			
Recessive model	TT + TC	1	0.255
	CC	1.165 (0.896–1.515)	
Dominant model	TT	1	0.418
	CC + TC	1.140 (0.830–1.566)	
Additive model	TT	1	
	TC	1.080 (0.770–1.514)	0.655
	CC	1.231 (0.861–1.761)	0.254
Multiplicative model	C allele	1.114 (0.935–1.327)	0.228

### Association of SNPs with lung cancer risk in multivariate analysis

Next, we evaluated independent risk factors of lung cancer using multivariate analysis. By incorporating environmental and lifestyle parameters, we found that in the recessive model, *Musashi-1* rs2522137 was still significantly associated with lung cancer. These environmental and lifestyle parameters included lower BMI, family history of cancer, prior diagnosis of COPD, pneumonia or pulmonary tuberculosis, occupational exposure to pesticide, occupational exposure to gasoline or diesel, heavier smoking, and exposure to heavier cooking emission (**Table 4**). These data suggest that the *Musashi-1* rs2522137 GG genotype is a significant genetic risk factor for lung cancer.

**Table:** **Table 4.** Multivariate risk model with adjusted odds ratios and 95% confidence intervals.

Risk factors	Exp (B)	95% C.I.	P value
Occupational exposure to pesticide			
Absence	1.00	Reference	0.000
Presence	3.390	(2.093–5.493)	
exposure to gasoline/diesel:			
Absence	1.00	Reference	0.012
Presence	4.653	(1.402–15.448)	
Smoking	1.032	(1.024–1.041)	0.000
Cooking emission(Total dish-years)			0.001
≤50	1.00	Reference	
51–100	1.304	(0.934–1.819)	0.119
101–150	0.941	(0.608–1.457)	0.785
>150	3.375	(1.779–6.402)	0.000
COPD:			
History absence	1.00	Reference	0.000
History presence	3.775	(1.809–7.878)	
Pneumonia:			
History presence	1.00	Reference	0.021
History absence	0.369	(0.158–0.860)	
Pulmonary tuberculosis:			
History presence	1.00	Reference	0.022
History absence	0.428	(0.207–0.884)	
Family history of cancer:			
History absence	1.00	Reference	0.000
History presence	1.848	(1.338–2.553)	
BMI(kg/m2):			0.000
<18.5	1.00	Reference	
18.5–24	0.370	(0. 192–0. 714)	0.003
≥24	0.168	(0.086–0. 328)	0.000
rs2522137:			
TT+TG	1.00	Reference	
GG	1.926	(1.209–3.070)	0.006

****COPD: chronic obstructive pulmonary disease

### ROC analysis

**Figure 1:**
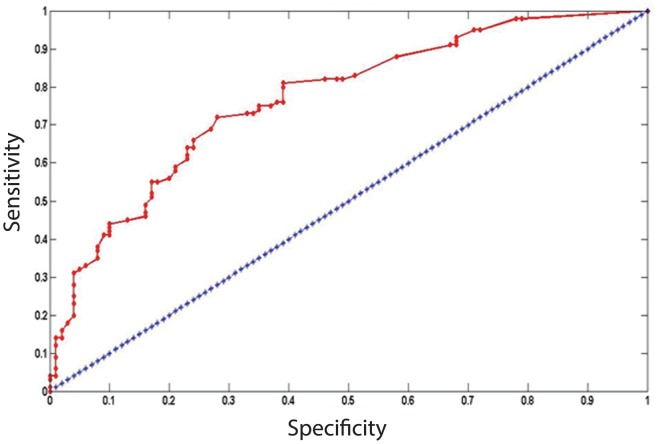
ROC plots for lung cancer risk prediction model. The ROC AUC was 0.7686. The straight line represented the ROC curve expected by chance alone.

The classification ability of the multivariate model was further evaluated using the area under ROC curve (AUC) and the optimal operating point (OPP). **Figure 1** shows the ROC curve derived from our model; AUC was calculated as 0.7686. Furthermore, the OPP was obtained when the cutoff was set at 0.47. The estimated false positive rates, true positive rates, and Youden index were determined to be 0.28, 0.72, and 0.44, respectively.

### Correlation between SNPs and lung cancer type

Finally, we looked for correlations between CSC SNPs and lung cancer type (squamous cell, adenocarcinoma, small cell) along with age at onset and gender of lung cancer. We did not observe statistically significant differences between these CSC SNPs and age or gender at the onset of lung cancer (**Tables 5**). In the pathology-stratified analysis, however, CD133 SNP rs2286455 was significantly correlated with lung cancer type (P = 0.048) (**Table 6**). No differences were observed between the remaining SNPs being considered with lung cancer type.

**Table 5:** Association of SNPs with gender and age from lung cancer patients.

Characteristic		Gender		*P* value	Age (yrs.)	*P* value
		M*	F*			
rs2286455	AA	29	17	0.184	58.87±10.18	reference
	GG	171	95		57.07±11.34	0.257
	GA	105	83		59.02±9.22	0.872
rs1342024	CC	99	65	0.806	58.85±9.95	reference
	GG	62	35		59.86±9.95	0.432
	GC	144	95		58.25±9.93	0.550
rs13959	CC	103	73	0.574	59.36±9.78	reference
	TT	58	31		60.15±9.84	0.541
	CT	144	91		57.79±10.03	0.112
rs2522137	GG	39	28	0.877	58.27±10.52	reference
	TT	135	84		58.07±10.15	0.885
	GT	131	83		59.62±9.50	0.331
rs17036526	CC	31	24	0.740	59.80±9.90	reference
	GG	139	85		58.90±9.08	0.547
	CG	135	86		58.36±10.76	0.338

*M, male; F, female.

**Table 6:** Association of SNPs with histology types from lung cancer patients.

Characteristic		Histology types				*P* value
		SQ*	AD*	SC*	OC*	
rs2286455	AA	78	93	70	25	0.048
	GG	10	16	18	2	
	GA	53	67	38	30	
rs1342024	CC	41	59	43	21	0.640
	GG	30	39	20	8	
	GC	70	78	63	28	
rs13959	CC	49	64	42	21	0.324
	TT	23	39	22	5	
	CT	69	73	62	31	
rs2522137	GG	21	25	18	3	0.361
	TT	65	69	59	26	
	GT	55	82	49	28	
rs17036526	CC	15	19	13	8	0.780
	GG	70	76	52	26	
	CG	56	81	61	23	

* SQ: squamous cell; AD, adenocarcinoma; SC, small cell; OC, other carcinomas.

## Discussion

Single nucleotide polymorphisms (SNPs) have been extensively examined in practically all cancer types in an effort to identify inherited cancer susceptibility genes and their interaction with environmental factors. Cancer stem cells (CSCs) play an important role in tumor initiation, metastases, and recurrence. We have examined the potential correlation between SNPs present in CSCs and the likelihood of lung cancer. This is particularly important since SNPs in CSC-directing genes could provide a genetic link to cancers that are particularly challenging to treat. While typical cancer therapies may eliminate most of the tumor mass, a small population of CSCs with the potential to repopulate the tumor may remain [5]. It is generally accepted that CSCs are characterized by the unique expression of cell surface molecules called CSC marker genes. CSC markers play an important part in the maintenance of self-renewal and resistance to apoptosis pathway activation in these cells. In this study, we obtained information to support the hypothesis that clinical outcome in lung cancer patients may be influenced by genetic variants of CSC marker genes.

The potential impact of CSC marker gene polymorphisms on lung cancer susceptibility has not been previously explored. In this study we took the advantage of a hypothesis-driven candidate gene approach [38]–[40] to identify potentially functional SNPs associated with histologically validated lung cancer. In contrast to genome-wide association (GWA) and quantitative trait locus (QTL) approaches, the candidate gene approach is economical and has rather high statistical power [38]. We focused on four CSC marker genes that have been used to isolate CSCs: CD133, ALDH1, EpCAM, and *Musashi-1* [41], [42]. Using the candidate gene approach, we selected a panel of SNPs in these CSC gene loci from SNP websites and peer-reviewed literature. SNPs identified to have high allele frequency were genotyped in 500 lung cancer cases along with 500 age-matched controls. Our results have identified the *Musashi-1* variant as an independent risk factor for lung cancer. It is also interesting to note that the *Musashi-1* rs2522137 genotype was still associated with lung cancer risk in a multivariate regression model that considered several environmental and lifestyle factors. Taken together, this study provides the first evidence to correlate the *Musashi-1* rs2522137 SNP variant with lung cancer.

Currently, we know very little about the detailed molecular mechanisms by which *Musashi-1* rs2522137 polymorphisms might contribute to lung cancer development. *Musashi-1* is an evolutionarily conserved RNA-binding protein that has profound implications in cellular processes, such as stem cell maintenance, nervous system development, and tumorigenesis. *Musashi-1* is highly expressed in many cancers, whereas in normal tissues, its expression is restricted only to stem cells. It is now clear that this RNA-binding protein is involved in cell asymmetric division and is required for the maintenance of stem cell identity [43]–[45]. Interestingly, *Musashi-1* mRNA transcript contains an 1811-bp long 3′-untranslated region (3′-UTR). The 3′-UTR of mRNA transcripts usually buries the target site for regulatory microRNA (miRNA). SNPs in the 3′-UTRs have been shown to have functional effects on control of mRNA stability and/or translational efficiency through the regulation of miRNA. The binding of miRNAs to the 3′-UTRs may play an important overall role in gene expression.

The 3′-UTR of mature *Musashi-1* mRNA is potentially targeted by several tumor suppressor miRNAs, including miR-34a, -101, -128, -137 and -138 [46]. In addition, the *Musashi-1* mRNA 3′-UTR contains several AU- and U-rich sequences that are targeted by an evolutionarily conserved RNA-binding protein HuR [47]. HuR is a member of the Hu/ELAV (embryonic lethal abnormal vision) family, which is highly expressed in tumor tissue and enhances tumorigenesis by interacting with a subset of mRNAs that encode proteins in the regulation of cell proliferation, cell survival, angiogenesis, invasion, and metastasis [48], [49]. Using the SNPinfo website (http://snpinfo.niehs.nih.gov/), we found that the *Musashi-1* 3′-UTR also buries potential target sites for miRNAs hsa-miR-1275, hsa-miR-1285, hsa-miR-483-5p, hsa-miR-486-3p, hsa-miR-612, and hsa-miR-625. It is worthwhile noting that *Musashi-1* rs2522137 is located within these miRNA and HuR binding sites. Future studies are needed to delineate whether rs2522137 variants may affect the binding of these regulatory miRNAs and HuR protein. Presumably, the *Musashi-1* rs2522137 GG variant may interfere with the binding of miRNAs and HuR factor, thus increasing the stability of *Musashi-1* mRNA. If true, this mechanism could provide the basis for the *Musashi-1* rs2522137 variant to maintain self-renewing lung cancer stem cells.

SNPs represent inherited genetic variations that occur during the lifetime of an individual. It is well known that non-genetic risk factors, such as age, history of lung disease, and smoking history are also very important and can be combined to develop risk-based models of cancer. Robert et al [50], [51] have suggested that SNPs need to be combined with other risk variables to identify individuals who are most susceptible to developing lung cancer. Similarly, the Liverpool Lung Project risk model improves its predictive capability of lung cancer by adding a marker SNP (rs663048) in the SEZ6L gene [52], [53]. In this study, we identified a correlation between lung cancer and a specific SNP within a CSC marker gene. Environmental and lifestyle factors included in this analysis, such as occupational exposure to pesticide, occupational exposure to gasoline/diesel prior diagnosis of pulmonary tuberculosis, and cooking emission, provide a similar correlation relative to an age-matched control population.

In summary, this study revealed a significantly increased risk of lung cancer for the CSC marker *Musashi-1* rs2522137-GG compared with -TT and -TG SNPs in a Chinese population. The ROC AUC of our model was 0.7686, indicating the potential to identify high-risk individuals in the Chinese population by focusing on information that can be readily obtained in the primary care setting. Finally, this lung cancer risk prediction model discriminated between high- and low-risk individuals. Further studies are needed in larger cohorts of unselected cases and controls to further validate and extent these initial observations.
